# Dimensional accuracy of dental impression materials evaluated using a standardized CAD based method across materials tray types and techniques

**DOI:** 10.1038/s41598-026-58096-8

**Published:** 2026-06-15

**Authors:** Christoph Wassermann, Nico Rosenberger, Marcel Gerhardt, Johannes Schrenker, Marc Schmitter, Christian Hoehne

**Affiliations:** 1 Department of Prosthodontics, University of Wuerzburg, Pleicherwall 2, 97070 Wuerzburg, Germany; 2 Private Practice, Zahnaerzte Lindlar und Frielingsdorf, Rheinstraße 3, 51789 Lindlar, Germany

**Keywords:** Dental impression, Custom tray, Stock tray, Impression materials, Additive manufacturing, Engineering, Health care, Materials science, Medical research

## Abstract

In this in vitro study, a CAD-based test workflow was established to evaluate the dimensional accuracy of conventional dental impression materials under standardized laboratory conditions. Ninety double-mix impressions were made from a rigid metal reference arch with four reference spheres using three elastomeric materials: vinyl polysiloxane (VPS), polyether (PE), and vinyl siloxanether (VSE). Guided impressions and freehand impressions were performed with both stock and custom trays. Impressions were cast in type IV dental stone, digitized with a laboratory scanner, and analyzed by comparing four linear inter-sphere distances with the reference model. Non-parametric statistics were applied. VPS and PE showed significantly lower global deviations than VSE, while VPS and PE did not differ significantly from each other. No significant differences were found between guided and freehand impressions when using stock trays. In the freehand-only comparison, stock and custom trays did not differ significantly; a borderline difference in the full cohort should be interpreted cautiously because of the partially crossed design. All measured deviations remained below the 1.5% linear-change threshold specified in DIN EN ISO 4823. This is the German adoption of a European and international standard for dentistry, specifically elastomeric impression materials. Within the limitations of this in vitro stone-cast workflow and linear-distance analysis, material selection had the strongest effect on global dimensional accuracy.

## Introduction

Accurate conventional impressions remain essential for many prosthodontic workflows, particularly when a stone-cast pathway is used. The dimensional behavior of impression materials can influence the geometric fidelity of the resulting cast and thereby affect subsequent restorative procedures. Recent systematic reviews have highlighted a fundamental limitation: the lack of standardized test strategies severely prevents comparability across studies investigating impression accuracy^1–4^. Study designs vary widely in reference model geometry, measurement methodology, and impression protocol, making it difficult to draw definitive conclusions about material or technique superiority^1,3^. Standardized metrological workflows are therefore needed to enable reproducible evaluation approaches, particularly in the context of digital superimposition and CAD-based deviation analysis^5,6^.

The present study introduces a CAD-based in-vitro test framework combining a rigid metal reference model with integrated reference spheres, a standardized cast workflow, and digital metrology based on inter-sphere distances. The framework was applied to compare three elastomeric materials. Vinyl polysiloxane (VPS), polyether (PE), and vinyl siloxanether (VSE) were tested across guided and freehand impression protocols with stock and custom trays.

The study tested the hypotheses that (H0a) no differences in global dimensional accuracy exist among the three materials; (H0b) no differences exist between stock and custom trays within the freehand-only comparison; and (H0c) no differences exist between guided and freehand impressions when using stock trays.

## Methods

### Study design

**Fig. 1:**
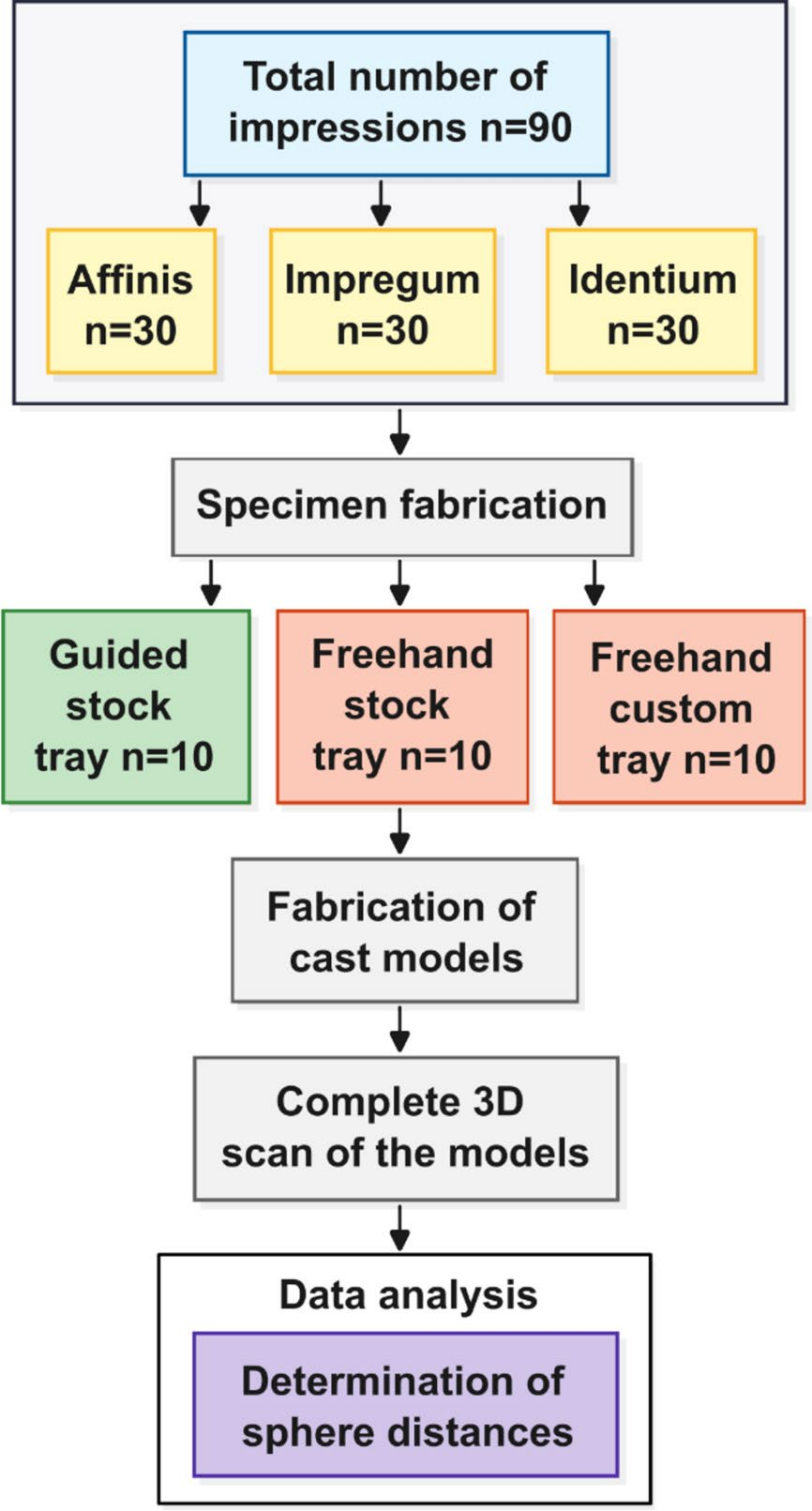
Schematic diagram of the study design.

Ninety double‑mix impressions of a standardized metal reference model were produced. Three elastomeric materials and three impression modes were used in a partially crossed in vitro design containing three materials and three experimental groups: guided stock tray (GS), freehand stock tray (FS), and freehand custom tray (FC). Each material-mode combination contained ten specimens. Materials were Affinis (VPS; Coltène, Langenau, Germany), Impregum Penta (PE; Solventum Germany, Kamen, Germany), and Identium (VSE; Kettenbach, Eschenburg, Germany) (Fig. 1). Because guided impressions were not combined with custom trays, the effects of tray type and impression method could not be evaluated as fully independent factors across the entire dataset. Accordingly, comparisons of tray type were restricted primarily to the freehand-only cohort (FS vs. FC), and comparisons of impression method were restricted to stock trays (GS vs. FS). The order of impression fabrication was randomized using a computer-generated sequence prepared before data collection to reduce temporal drift and operator learning effects. Data collection extended over more than one year.

### Reference model

**Fig. 2:**
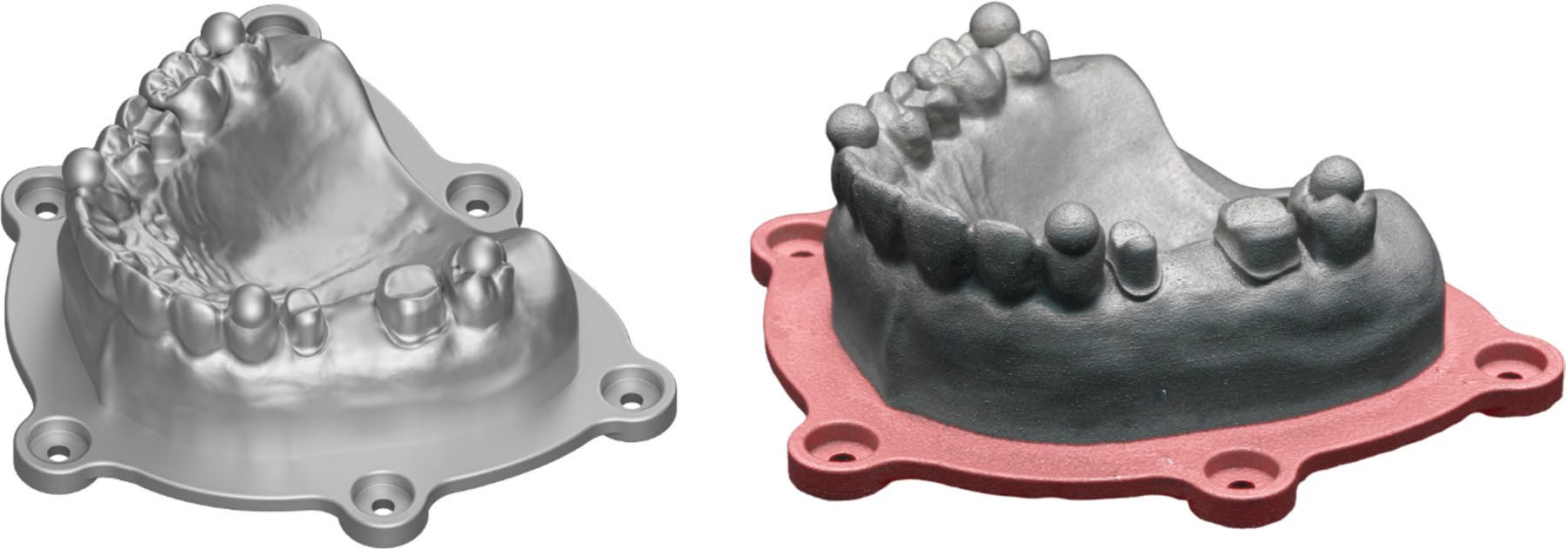
Refined construction of the reference model in CAD (left). SLM printed metal model with the base socket highlighted in red (right).

The reference model was derived from a KaVo maxillary study arch (KaVo, Biberach an der Riß, Germany) in which the right second premolar was removed. The right first premolar, right first molar and left second premolar were prepared with clinically representative geometries consisting of chamfer and partial‑coverage preparations. The model was digitized via impression‑to‑stone‑to‑scan workflow using an InEos X5 laboratory scanner (Dentsply Sirona, Bensheim, Germany), refined in CAD software (Autodesk Meshmixer and Autodesk Fusion; Autodesk, Inc. San Francisco, CA, USA), (Fig. 2 left) and equipped with a base socket for the guided system (Fig. 2 right in red). Four reference spheres (Ø 6 mm) were virtually placed on the right and left second molars and canines. They were positioned to avoid interference with surface analyses while minimizing risk of plastic deformation during repeated impressions.

The digital model was manufactured by BEGO (BEGO GmbH & Co. KG, Bremen, Germany) using selective laser melting (SLM) from Wirobond C+ cobalt-chromium alloy (BEGO GmbH & Co. KG, Bremen, Germany) at 2 mm wall thickness to prevent deformation across the study.

## Impression trays

Stock impressions (groups GS and FS) used a stainless‑steel Rimlock tray (Algilock OK/BS 3, size XL; Hager & Werken, Duisburg, Germany). Custom trays (group FC) were designed in Autodesk Fusion with a uniform 3 mm offset to all model surfaces including the spheres, 2 mm wall thickness, no perforations, a frontal handle, and three guidance loops which were between the middle incisors and the left and right second molars. Custom trays were printed on a Form3B (Formlabs Inc., Sommerville, USA) using a dedicated custom‑tray resin (Formlabs Custom Tray Resin, RS-F2-CTBL-01, Formlabs Inc., Sommerville, USA). After printing, all trays were washed in isopropanol (Form Wash, Formlabs Inc., Sommerville, USA), post‑cured at 60 °C (Form Cure, Formlabs Inc., Sommerville, USA) for 30 min, and internally air‑abraded with 50 μm Al₂O₃ (Cemat-NT4, Wassermann, Hamburg, Germany) to enhance micromechanical retention. Tray adhesives were applied according to the impression material: a universal adhesive for VPS (Coltène, Langenau, Germany), a vinylsiloxanether adhesive for VSE (Kettenbach, Eschenburg, Germany), and a polyether adhesive for PE (Solventum Germany, Kamen, Germany).

## Guided impression system

**Fig. 3:**
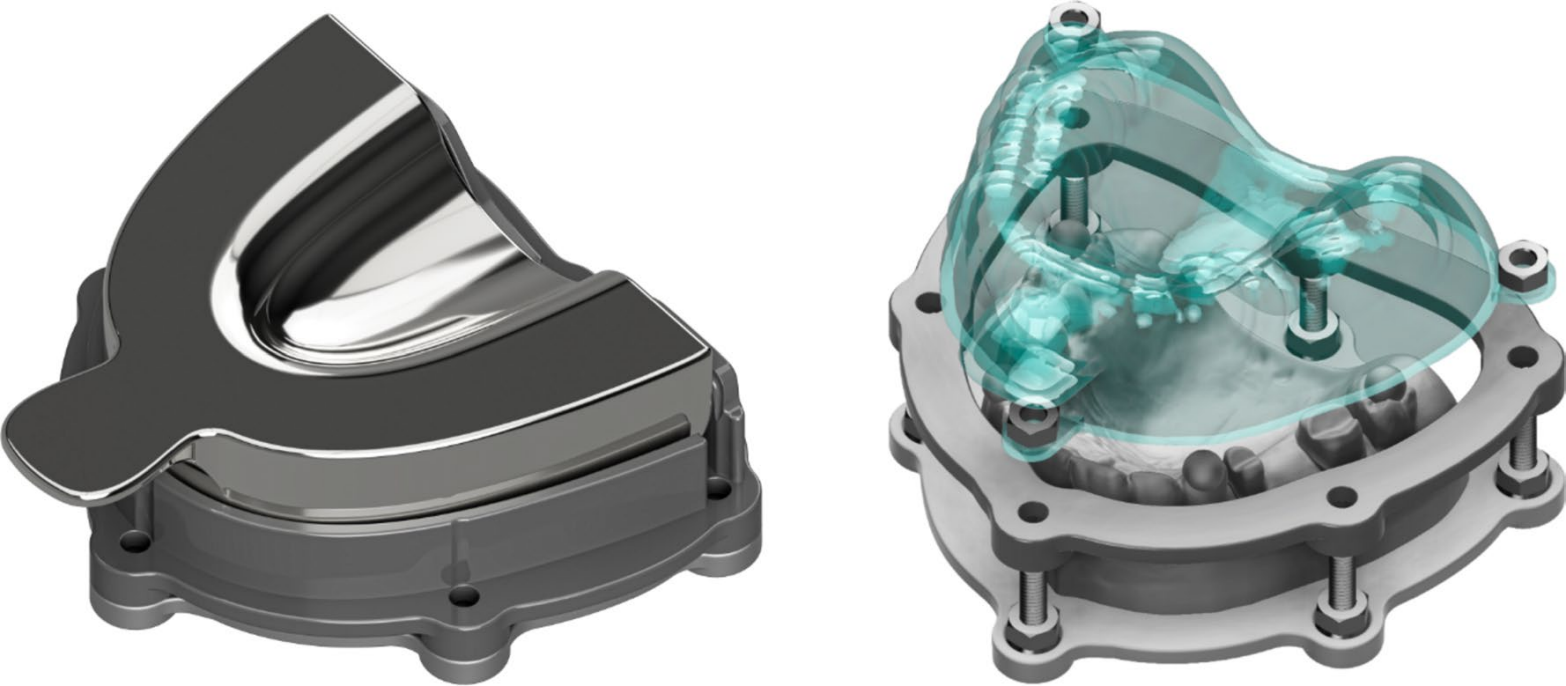
Visualization of the guided impression system with a stock tray.

**Fig. 4:**
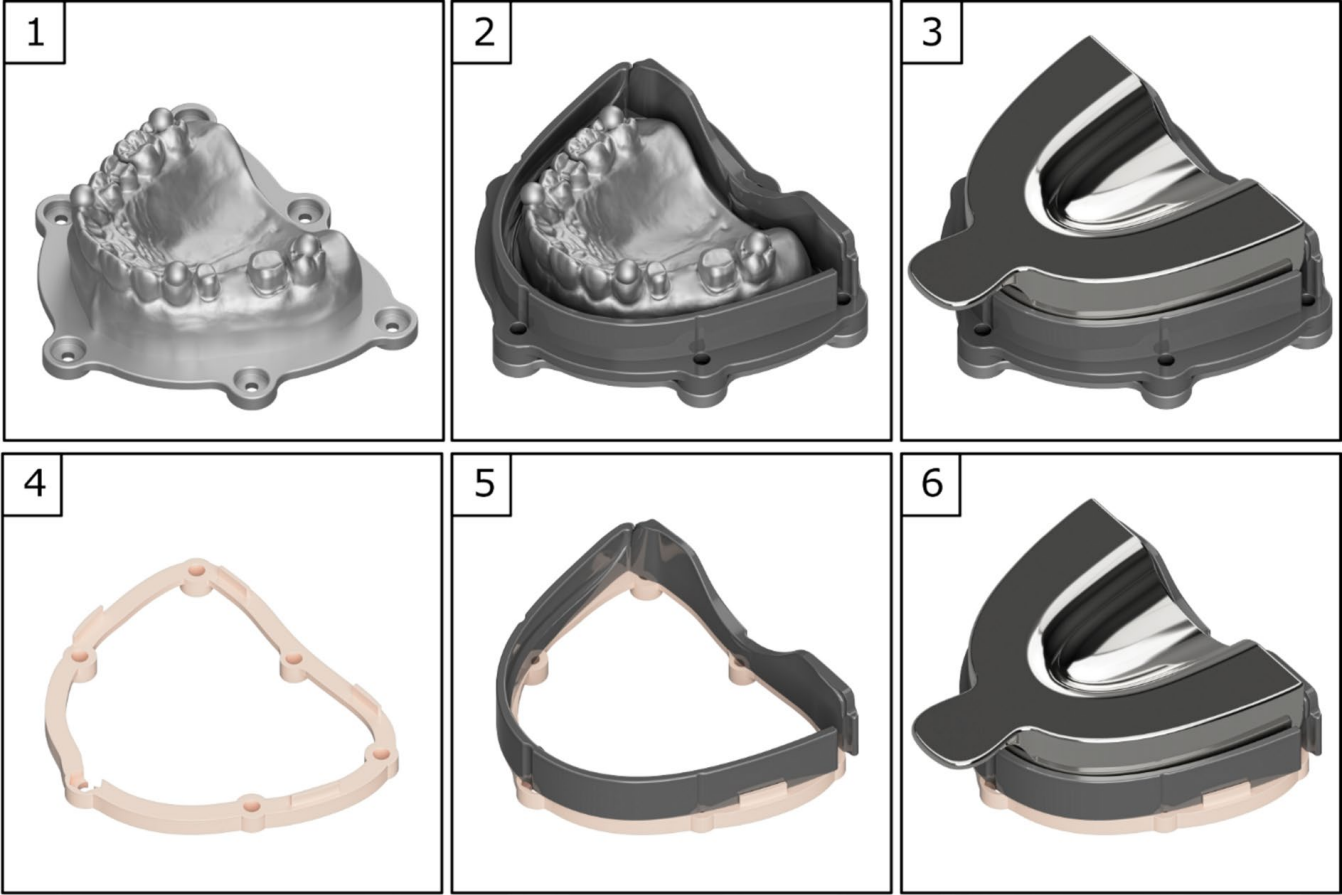
Display of the process of the guided impression (1–3) and the preparatory steps for the cast fabrication (4–6).

**Fig. 5:**
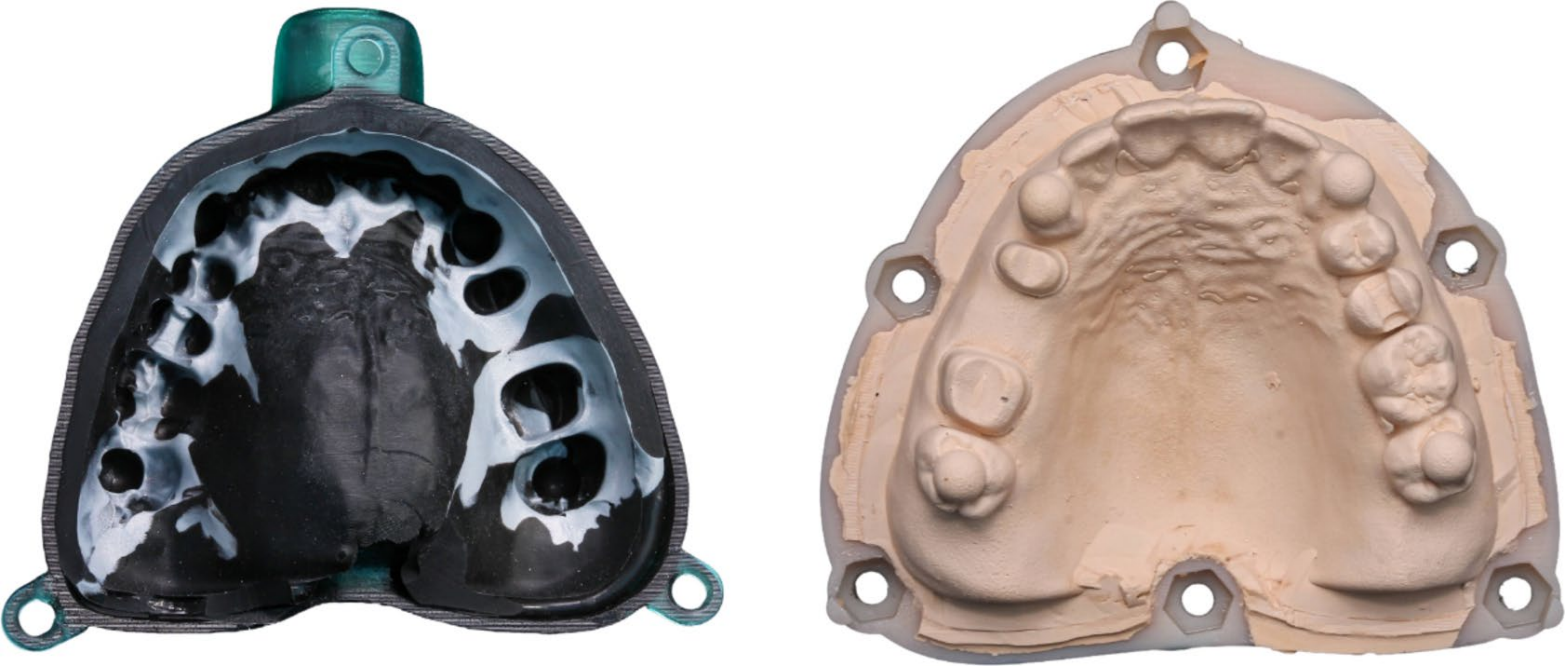
The impression with Affinis (left). The cast after guided demoulding (right).

**Fig. 6:**
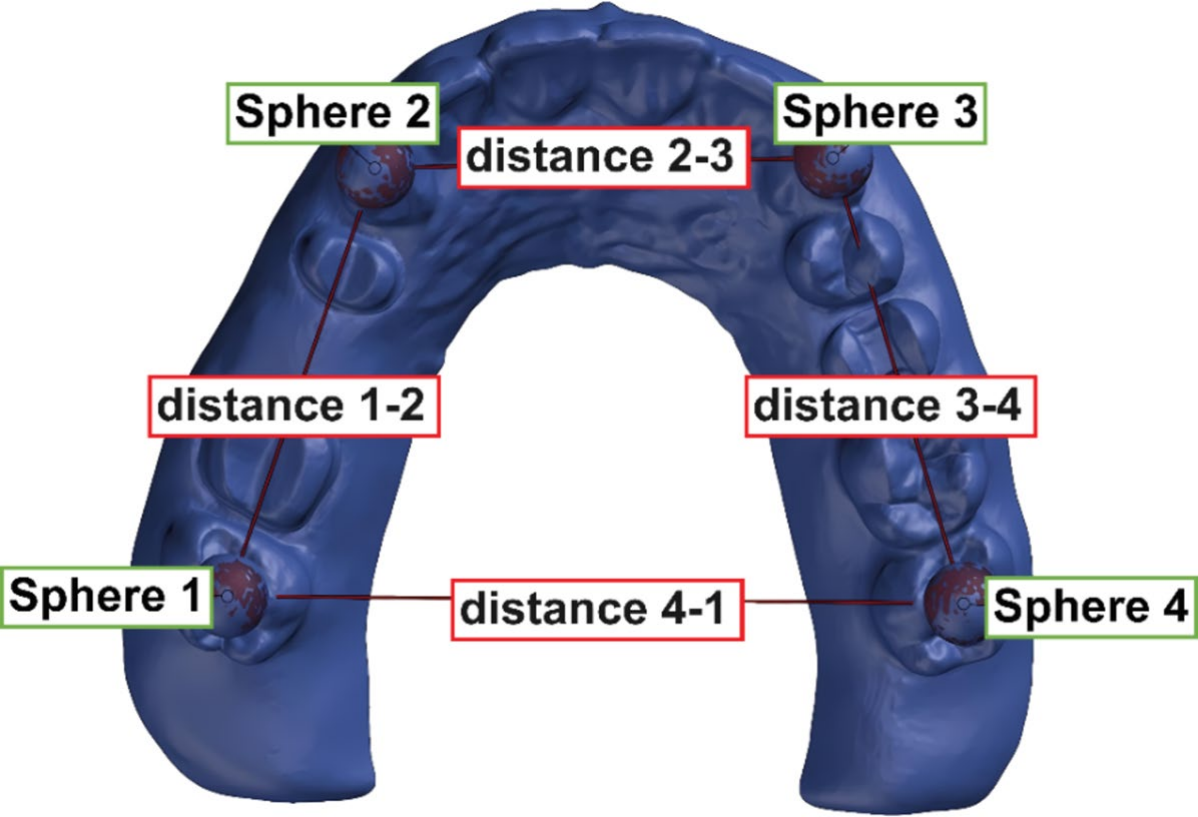
Digitized reference model with marked spheres (green frames) and distances (red frames).

A purpose‑built guidance and demoulding device was developed for stock and custom trays to standardize tray positioning and removal (Fig. 3). The system contained: (1) a base socket integrated within the reference model (Fig. 4.1), providing a vertical stop and six threaded attachment points; (2) an adapter ring for the impression tray, ensuring guided seating and enabling screw‑assisted axial demoulding (Fig. 4.2); (3) on this the stock tray can be placed (Fig. 4.3); (4) a plaster adapter for standardized and the sleeve-type adapter (left) and a custom tray with the adapter ring (right). cast pouring (Fig. 4.4); (5) for stock trays, a sleeve‑type adapter with raised circumferential walls accommodating the broader tray geometry (Fig. 4.5); (6) on this the tray with the impression can be placed for casting out with dental stone (Fig. 4.6). Six countersunk screws (M4 × 16 mm, stainless steel A2) engaged threaded inserts in the adapter, transmitting axial force to separate the set impression from the reference without leveraging. This near‑axial removal path was designed to minimize peel forces and bending‑induced distortion.

## Impression protocol

All impressions followed the double‑mix technique: low‑viscosity material was syringed onto spheres, preparations, undercuts, and fissures while high‑viscosity material was simultaneously dispensed into the tray using a Pentamix 3 mixing unit (Solventum Germany, Kamen, Germany). Material‑specific tray adhesives were applied and dried before each impression (Table 1). Because the reference model was used at room temperature rather than intraoral temperature, two additional minutes were added to the manufacturers’ specified intraoral setting times to reduce the risk of premature removal under lower-temperature laboratory conditions. A thin layer of petroleum jelly was applied only to non-critical non-measurement surfaces to facilitate demoulding.

**Table 1:** Impression materials and corresponding adhesives.

Material	High‑viscosity	Low‑viscosity	Adhesive
Affinis (VPS) (Coltene)	System 360 Heavy Body	Precious Light Body	Universal Adhesive (Kulzer)
Impregum (PE) (Solventum)	Impregum Penta	Permadyne Garant	Polyether Adhesive (Solventum)
Identium (VSE) (Kettenbach)	Identium Heavy	Identium Light	Identium Adhesive (Kettenbach)

## Cast fabrication

Impressions were poured according to the manufacturer’s instructions (Fig. 5), using type IV dental stone (Fujirock EP golden brown; GC Europe, Leuven, Belgium) at a standardized 100 g / 20 ml powder‑to‑water ratio. Mixing contained 15 s manual spatulation followed by 45 s vacuum mixing (Wamix, Wassermann Dental, Hamburg, Germany). Stone was vibrated into the impression (D‑R 644, Harnisch + Rieth, Winterbach, Germany), filling to just above the adapter’s outer wall. Casts were stored undisturbed for at least one hour before guided demoulding (Fig. 5 right).

### Digitization and data preparation

All casts were scanned with the same calibrated InEos X5 laboratory scanner and exported as STL files. Data preparation involved trimming to the tooth‑crown level in Autodesk Meshmixer, removing surface artifacts (air bubbles, stone pearls), remeshing all files uniformly in Autodesk Fusion, and correcting surface‑normal orientation in GOM Inspect (Version 2018, GOM GmbH, Braunschweig, Germany). Test files were pre‑aligned to the reference using initial prealignment followed by local best‑fit registration in GOM Inspect.

## Metrology

In GOM Inspect, ideal virtual fitting spheres were generated on the digitized spherical reference areas of each cast after standardized trimming, remeshing, and alignment. Sphere centers were thus derived automatically from the fitted geometry rather than by manual visual point selection. Four inter-sphere linear distances were then calculated: S1 (distance 1–2), S2 (distance 2–3), S3 (distance 3–4), and S4 (distance 4–1), summing to a total reference length of 152.47 mm (Fig. 6). Absolute deviations from the reference distances were computed for each specimen. Deviations were analyzed both as absolute values and with preserved sign to distinguish contraction from expansion. The operator performing the digital measurements was blinded to material and group allocation during metrology analysis.

### Statistical analysis

No a priori formal sample size calculation was performed. The sample size of *n* = 10 per experimental cell was selected for feasibility and with reference to comparable in vitro studies on impression accuracy. This should be considered when interpreting null findings and small between-group differences. Descriptive statistics are reported primarily as median (Md) and interquartile range (IQR). Normality was assessed using the Kolmogorov-Smirnov and Shapiro-Wilk tests. Because most distributions were non-normal, non-parametric methods were used. Omnibus comparisons were performed using the Kruskal-Wallis test, followed by Mann-Whitney U tests with Bonferroni correction for pairwise comparisons where appropriate. Exact p-values are reported throughout. The significance level was set at α = 0.05 and adjusted for multiple pairwise comparisons where applicable. Analyses were performed using IBM SPSS 27 (IBM Corp., Armonk, NY, USA) and Microsoft Excel (Microsoft Corporation, Redmond, WA, USA), with biostatistical consultation from the Institute of Clinical Epidemiology and Biometry at the University of Wuerzburg.

## Results

All 90 impressions and corresponding casts were analyzed without exclusion. Deviations are presented as summed total-distance deviations across the four inter-sphere segments, referenced to a total nominal length of 152.47 mm, and as individual segment deviations. Because the study design was partially crossed, comparisons of tray type were performed primarily within the freehand-only cohort (FS vs. FC), whereas comparisons of impression method were restricted to stock trays (GS vs. FS).

### Effect of impression material (*n* = 90)

Affinis (VPS) showed the lowest median total‑distance deviation (Md 124 μm; 0.08%), followed by Impregum (PE; Md 132 μm; 0.09%) and Identium (VSE; Md 201 μm; 0.13%). Affinis and Impregum did not differ significantly from each other (*p* = 0.024) but were significantly more accurate than Identium (*p* < 0.001). Impregum exhibited the greatest variability (range 393 μm) and was the only material producing consistently negative deviations, indicating a shrinkage tendency (Table 2).

**Table 2:** Total distance deviations by impression material (absolute values, *n* = 90).

Md ± IQR (µm)	Affinis (VPS)	Impregum (PE)	Identium (VSE)
	124 ± 51	132 ± 75	201 ± 89
Md ± IQR (%)	0.08 ± 0.03	0.09 ± 0.05	0.13 ± 0.06
p-values	VPS vs. PE 0.024	PE vs. VSE < 0.001	VPS vs. VSE < 0.001

Individual‑segment analysis confirmed these trends: Identium showed significantly higher deviations on all four segments (S1–S4; *p* < 0.001), with the sole exception of S1 where the Impregum-Identium difference fell below significance after Bonferroni correction (*p* = 0.04 vs. threshold 0.017). No significant differences between Affinis and Impregum were detected on any segment, except S3 in the signed‑value analysis (*p* < 0.001). Impregum consistently generated the highest variability and the most negative deviations across all segments. Bootstrap-derived 95% confidence intervals (CI) for median total-distance deviations were 104–141 μm for Affinis (VPS), 112–148 μm for Impregum (PE), and 187–232 μm for Identium (VSE). The confidence intervals of VPS and PE showed substantial overlap, whereas VSE exhibited consistently higher values with minimal overlap. Effect size analysis using Cliff’s delta demonstrated a small difference between VPS and PE (δ = -0.18), but large to very large effects for comparisons involving VSE (PE vs. VSE: δ = -0.56; VPS vs. VSE: δ = -0.78), indicating substantially higher dimensional deviations for VSE.

### Effect of tray type (*n* = 60 and *n* = 90)

In the freehand-only cohort (*n* = 60), no significant differences were found between custom and stock trays in either absolute (*p* = 0.174) or signed analyses (*p* = 0.554). Custom trays showed a tendency toward lower median deviation but higher variability (Table 3). In the full cohort (*n* = 90), stock trays showed a slightly lower median absolute deviation than custom trays (Md 136 μm vs. 167 μm; *p* = 0.049). However, this borderline finding was not supported by the signed-value analysis (*p* = 0.05) and should be interpreted cautiously because of the partially crossed design and the small effect size. In the freehand-only cohort, median total-distance deviation was 144 μm (95% CI: 132–152 μm) for stock trays and 167 μm (95% CI: 132–197 μm) for custom trays. The confidence intervals showed substantial overlap. Effect size analysis indicated a small difference between tray types (Cliff’s δ = -0.20), supporting the absence of a meaningful difference. In the full cohort, median deviation was 136 μm (95% CI: 124–146 μm) for stock trays and 167 μm (95% CI: 132–197 μm) for custom trays. Although the medians differed slightly, confidence intervals overlapped substantially. Effect size analysis revealed a small to approaching moderate effect (Cliff’s δ = -0.26), indicating that the observed difference was limited in magnitude.

**Table 3:** Total distance deviations by tray type (absolute values).

Md ± IQR (µm)	Custom (*n* = 60)	Stock (*n* = 60)	Custom (*n* = 90)	Stock (*n* = 90)
	167 ± 77	144 ± 59	167 ± 77	136 ± 80
Md ± IQR (%)	0.11 ± 0.05	0.09 ± 0.04	0.11 ± 0.05	0.09 ± 0.05
p-value	Custom vs. Stock *p* = 0.174		Custom vs. Stock *p* = 0.049	

### Effect of impression method (*n* = 30)

Because guided impressions were performed only with stock trays, the comparison of impression method was restricted to guided stock (GS) versus freehand stock (FS) impressions (*n* = 30 per group). No significant differences were detected between GS and FS in either absolute or signed analyses (*p* = 0.225). Freehand stock impressions showed a numerically slightly higher median deviation, but the difference was not statistically significant (Table 4). For impression method, median total-distance deviation was 144 μm (95% CI: 132–152 μm) for freehand impressions and 122 μm (95% CI: 102–143 μm) for guided impressions. The confidence intervals overlapped considerably. Effect size analysis indicated a small difference (Cliff’s δ = 0.17), confirming that the difference between guided and freehand impressions was negligible.

**Table 4:** Total distance deviations by impression method within stock trays only (absolute values).

Md ± IQR (µm)	Freehand stock (*n* = 30)	Guided stock (*n* = 30)
	144 ± 49	121 ± 79
Md ± IQR (%)	0.09 ± 0.03	0.08 ± 0.05
p-value	Freehand stock vs. Guided stock *p* = 0.225	

## Discussion

### Principal findings

This study established and applied a standardized CAD‑based in vitro test model with four reference spheres and a guided impression system. It was used to evaluate three elastomeric dental impression materials under controlled conditions. The material factor had the strongest effect on global dimensional accuracy: Identium (VSE) showed significantly larger total‑distance deviations than both Affinis (VPS) and Impregum (PE), which performed comparably. Across all secondary comparisons, confidence intervals showed substantial overlap and effect sizes were small (|δ| ≤ 0.26), indicating that tray type and impression method had only minor influence compared to the strong material effect. Within the present in vitro stock-tray comparison, guided and freehand impressions did not differ significantly in global inter-sphere dimensional deviations. This finding should not be generalized to custom trays or to clinical intraoral performance, because the guided and custom tray condition was not tested. Also, clinically relevant factors such as saliva, restricted access, soft tissues, and patient movement were absent.

### Comparison with previous literature

The observed superiority of VPS and PE over VSE aligns with prior reports suggesting that vinyl siloxanether materials may exhibit greater cumulative distortion, particularly across longer spans due to their viscoelastic properties^7,8^. The comparable performance of VPS and PE is also consistent with the established literature, where both materials are generally regarded as highly accurate, although with different consistency characteristics^9,10^. The shrinkage tendency and higher variability observed for polyether correspond to known polymerization behavior described in prosthodontic studies^11^. With regard to tray design, the present findings do not fully support the conventional assumption that custom trays inherently improve accuracy. Previous studies have highlighted tray rigidity as a critical determinant, suggesting that metal stock trays may resist deformation more effectively than resin-based custom trays^12^. Finally, the lack of detectable differences between guided and freehand impressions contrasts with expectations that positioning aids improve accuracy. Comparable literature using similar full-arch reference systems is largely lacking, underlining the novelty of the present approach.

### Mechanistic interpretation

The findings can be interpreted primarily through material-specific viscoelastic and polymerization behavior. VPS demonstrated the most stable and consistent dimensional performance, reflecting its well-documented polymerization stability and low shrinkage^9,10^. In contrast, PE exhibited greater variability and a systematic contraction tendency, likely due to its hydrophilic and polymerization characteristics^11^. The comparatively higher deviation observed with VSE may reflect a trade-off inherent in hybrid material design, where combining properties of VPS and PE introduces more complex deformation behavior over extended spans^7,8^. The limited influence of tray type and guidance suggests that, under idealized laboratory conditions, material behavior outweighs procedural factors. The absence of differences between guided and freehand techniques likely reflects the lack of intraoral constraints. These are restricted access, soft tissues, and operator variability, which are known to amplify positioning errors clinically^13^.

### Methodological implications

A key contribution of this study is the introduction of a standardized CAD-based evaluation framework, addressing a major limitation identified in previous literature: the lack of comparability across studies due to heterogeneity in models and measurement methods^1–4^. The use of reference spheres with automated sphere-fitting and inter-sphere distance analysis provides a reproducible and operator-independent metric of global dimensional change. The integration of a guided impression and demoulding system further enhances process standardization and reduces technique-related variability. However, the reliance on linear measurements highlights a methodological trade-off: while robust and reproducible, this approach does not capture full-field deformation patterns that are increasingly evaluated using 3D surface deviation analyses^5,14^.

### Methodological value vs. clinical value

Te main value of the present work is methodological rather than clinical. The proposed workflow offers a standardized and transparent approach for comparing global dimensional behavior across materials and selected tray and method conditions in vitro. At the same time, the chosen metric with four inter-sphere distances captures global dimensional deviation only. However, full-arch studies demonstrate that accuracy is strongly influenced by scanning strategy, measurement approach, and cumulative distortion over longer spans^15,16^. Also, this does not provide information on full-surface distortion patterns, which are increasingly evaluated using full-field 3D deviation analyses based on digital superimposition workflows^5,14^. Consequently, the present findings should be interpreted primarily as evidence of comparative dimensional behavior within a controlled laboratory model and not as direct proof of clinical superiority, restoration fit, or intraoral performance. Clinical relevance will require further investigation under simulated and real intraoral conditions, ideally complemented by full-field 3D surface analyses and marginal-fit assessments.

### Strengths and limitations

Strengths include the robust NEM reference model, which has withstood up to 270 impressions without any detectable deformation. The model was tested before and after the impressions and no differences were detectable regarding scanner or model accuracy. Another strengths are the comprehensive standardization of all process steps, including material mixing, casting, curing, scanning, software configuration, the randomized execution sequence, and the CAD-based metrology pipeline with reproducible sphere fitting and distance measurements. The in vitro design enabled isolation of material and technical effects free from confounding clinical variables such as saliva, sulcular bleeding, and patient movement^13^.

Limitations inherent to the in vitro approach include the absence of soft‑tissue simulation, moisture, and temperature gradients present in clinical conditions. The focus on linear inter‑sphere distances provides a robust global metric but does not capture local surface deviations, marginal‑gap accuracy at preparation boundaries, cusp deformation, or full-surface warpage. The sample size of *n* = 10 per group is consistent with comparable in vitro studies^17,18^ but no a priori formal sample size calculation was performed, so the study may have been underpowered for small effects. Non-critical, non-measurement surfaces were lightly coated with petroleum jelly to facilitate demoulding. Although application was minimized and kept away from the reference spheres and prepared surfaces, a minor influence on material flow cannot be fully excluded. The guidance framework was technically adaptable to custom trays, but the guided and custom tray condition was not included in the present study design. Therefore, this was acknowledged as a missing experimental cell. Impression method and tray type could not be evaluated as fully independent factors across the full dataset. The CAD-based workflow was designed to ensure standardization. However, formal metrological validation was not performed. This includes repeated scanning, intra- and inter-operator reproducibility testing, sphere-fitting repeatability, and measurement uncertainty analysis. The addition of two minutes to the manufacturers’ setting times were standardization choices that may have influenced results to a limited extent.

### Future work

Ongoing sister studies will complete the missing guided and custom tray condition, which would require 30 additional impressions (10 per material) to balance the present 3 × 2 × 2 design. Beyond that, further studies are examining additional tray variables such as perforation design and wall thickness, incorporate full-field 3D surface analyses, and test the workflow under simulated or clinical conditions.

## Conclusions

- A novel, CAD-based in vitro test workflow using a rigid metal reference model and digital inter-sphere metrology was established for standardized comparative assessment of global dimensional deviations.
- Material selection had the strongest effect on global dimensional accuracy: VSE showed significantly larger deviations than VPS and PE, whereas VPS and PE performed comparably.
- PE showed higher variability and a tendency toward negative deviations, whereas VPS showed the most consistent performance.
- Within the stock-tray comparison only, no significant differences were found between guided and freehand impressions under controlled laboratory conditions.
- In the freehand-only comparison, stock and custom trays did not differ significantly; any broader interpretation is limited by the partially crossed study design.
- All measured deviations remained below the DIN EN ISO 4823 linear-change threshold; however, this does not by itself establish clinical acceptability.
- The findings are limited to an in vitro stone-cast workflow and to global linear-distance measurements rather than local marginal-fit or full-surface deviation analysis.
- Extension to full‑field 3D metrics, clinical scenarios, and additional tray configurations will refine the test framework and material recommendations.

## Data Availability

The datasets generated and analyzed during the present study are available from the corresponding author on reasonable request.
